# The Turkish version of the self-management efficacy questionnaire for patients with psoriasis: cross-cultural adaptation and psychometric evaluation

**DOI:** 10.1186/s12913-026-14487-1

**Published:** 2026-04-02

**Authors:** Emine Özer Küçük, Zuhal Candan Yamak, Gülay Keyik Kodalak, Esra Adışen

**Affiliations:** 1 Department of Internal Medicine Nursing, Gülhane Faculty of Nursing, University of Health Sciences, Ankara, Turkey; 2 Department of Dermatology and Venereology, Gazi University Health Research and Application Center, Ankara, Turkey

**Keywords:** Psoriasis, Self-management, Self-efficacy, Psychometrics, Nursing

## Abstract

**Background:**

Psoriasis is a chronic condition requiring sustained outpatient follow-up, patient education, and coordinated nursing care. Effective self-management is essential for supporting individualized patient education, guiding nursing care planning, and evaluating outpatient service outcomes; however, disease-specific tools to assess self-management efficacy are limited in Türkiye.

**Methods:**

This methodological study aimed to conduct the Turkish cross-cultural adaptation and psychometric evaluation of the Self-Management Efficacy Questionnaire for Patients with Psoriasis (SMEQ-PSO), originally developed by Sun et al. The study included 147 patients with psoriasis vulgaris attending a dermatology outpatient clinic. Content validity was assessed using expert evaluation, while construct validity was examined through exploratory and confirmatory factor analyses. Reliability was evaluated using Cronbach’s alpha, McDonald’s omega, and split-half methods.

**Results:**

Among 147 participants, 55.1% were male, with an average age of 45.16 (± 14.83) years. S-CVI/Ave was 0.97. EFA revealed a five-factor structure explaining 70.5% of the variance (KMO = 0.92). CFA showed acceptable model fit (χ²/df = 1.61, RMSEA = 0.07, CFI = 0.92). Cronbach’s α and McDonald’s Omega were both 0.95.

**Conclusions:**

The Turkish SMEQ-PSO is a valid and reliable instrument for assessing self-management efficacy among patients with psoriasis. Beyond individual assessment, the scale may support nursing-led care planning, targeted patient education, and evaluation of outpatient service delivery in chronic dermatological care settings.

## Background

Millions of people worldwide have psoriasis, a long-term inflammatory skin condition [1, 2]. Although its prevalence varies by region, it is estimated at approximately 4.4% globally, affecting 40.8 million people, and rises as high as 5.7% in East Asia [2, 3]. Clinically, it is characterized by silver-white scales developing on sharply defined, erythematous plaques and follows a course with remissions and exacerbations [4]. Psoriasis is not just a disease affecting the skin. It creates negative effects on quality of life comparable to chronic diseases such as ischemic heart disease, diabetes, and depression [5]. Visible skin lesions can lead to psychosocial problems such as social stigmatization, loss of self-confidence, anxiety, and depression in individuals [6]. The multidimensional effects of psoriasis necessitate the development of self-management skills [7, 8]. Self-management refers to an individual’s competence in managing their symptoms, treatment, physical and psychological outcomes, and daily life habits. Effective self-management requires individuals to monitor their condition and implement necessary cognitive, behavioral, and emotional strategies to maintain quality of life [9]. Given its chronic nature, psoriasis requires sustained outpatient follow-up and coordinated health service delivery to ensure optimal patient outcomes.

As the health professionals who maintain the most intensive and continuous contact with individuals living with chronic diseases, nurses are uniquely positioned to support self-management [10]. Indeed, research shows that 78% of nurses successfully carry out these interventions independently or as team leaders [11]. In their educator, consultant, and supportive roles, nurses enhance patients’ disease-related coping skills, strengthen treatment adherence, and improve quality of life. The positive effects of self-management education on reducing disease severity, increasing independence in daily activities, and improving psychosocial well-being have been demonstrated [8, 12, 13]. Additionally, nurse-supported education has been shown to improve self-management skills by increasing health literacy and disease knowledge [14]. Today, digital applications also allow nurses to continue this support online [15]. These findings underscore the nurse’s role in the effective management of psoriasis. From a health services perspective, nurses play a key role in ensuring continuity of care and optimizing resource utilization in outpatient dermatology settings.

However, for this support to be planned effectively, patients’ current self-management competencies in psoriasis must be objectively evaluated. Such an evaluation is important for developing personalized care strategies in clinical practices and measuring intervention effectiveness in research [16]. Current literature indicates that tools for assessing self-management skills among patients with psoriasis are limited [17]. This situation creates a significant gap in the personalization of care plans and in the evaluation of the effectiveness of intervention studies. Furthermore, such tools are essential for evaluating health service delivery and planning nursing-led interventions in chronic care settings.

Based on Bandura’s self-efficacy theory, the Self-Management Efficacy Questionnaire among Patients with Psoriasis (SMEQ-PSO) was developed to address this gap and to assess patients’ self-management efficacy [17]. In the original study, the 28-item scale with five dimensions demonstrated high internal consistency (Cronbach’s α = 0.93). However, there is no valid and reliable measurement tool for patients with psoriasis in Türkiye. Cross-cultural adaptation of measurement tools is essential not only for use in local populations but also for enabling international comparisons and contributing to global evidence in self-management research [18–20]. Accordingly, this study aimed to cross-culturally adapt the Turkish version of the SMEQ-PSO and evaluate its psychometric properties. This adaptation may contribute to improving outpatient care planning and health service evaluation in Turkish dermatological practice.

## Methods

### Design, setting, and sample

This methodological study was conducted between April and August 2024 among patients with psoriasis vulgaris admitted to the Dermatology Outpatient Clinic of the Gazi University Health Research and Application Center, using a consecutive sampling method. The inclusion criteria were: (1) aged 18 years or older; (2) diagnosed with psoriasis vulgaris within the previous year; (3) speaking Turkish; (4) having no communication barriers; and (5) voluntary agreement to participate in the study. Patients with cognitive impairment affecting comprehension, difficulty reading or writing in Turkish, and refusal to participate were excluded.

Sample size was determined based on the recommendation of at least five respondents per item for validity and reliability analyses [21]. With 28 items, the minimum required sample size was 140; the final sample included 147 participants.

### Data collection and instruments

Data were collected through face-to-face interviews during routine clinic visits. After being informed about the study’s purpose, eligible patients who provided written informed consent completed the questionnaires under supervision. Completion time was approximately 15–20 min.

#### Sociodemographic information form

After reviewing the literature, the researcher developed a 14-item form to collect information on participants’ sociodemographic characteristics (age, gender, marital status, educational background) and clinical features (disease duration, treatment approach) [12–14, 17, 22, 23].

#### Self-management efficacy questionnaire for patients with psoriasis (SMEQ-PSO)

The Self-Management Efficacy Questionnaire for Patients with Psoriasis (SMEQ-PSO) was originally developed and published by Sun et al. (2023) [17], and the present study aimed to conduct its Turkish cross-cultural adaptation and psychometric evaluation. Grounded in Bandura’s self-efficacy theory, this instrument evaluates patients’ confidence in managing their condition effectively. The SMEQ-PSO comprises 28 items across five dimensions: psychosocial adaptation self-efficacy (8 items), daily life management self-efficacy (8 items), skin care self-efficacy (5 items), disease knowledge management self-efficacy (4 items), and disease treatment management self-efficacy (3 items). Each item is rated on a 5-point Likert scale, from 1 (not confident at all) to 5 (completely confident). Total scores range from 28 to 140, with higher scores indicating better self-management efficacy. The internal consistency of the original scale was 0.93, and the subscales ranged between 0.71 and 0.90 [17].

### Translation and cultural adaptation of the scale

We followed the guidelines proposed by Beaton et al. for the translation and cultural adaptation of the scale into Turkish [18]. The six-step process included:

- *Step 1-Forward translation*: The original scale was independently translated into Turkish by five experts (two dermatology experts, two nursing faculty members, and one translator).
- *Step 2-Synthesis*: Researchers compared five translations and created a single Turkish form through consensus.
- *Step 3-Back translation*: Two independent native English-speaking translators, who were blind to the original instrument, back-translated the Turkish version into English.
- *Step 4-Expert panel*: A seven-member expert committee (two dermatologists, three nurses, one measurement specialist, and one language specialist) evaluated content validity using the Davis technique [24].
- *Step 5-Pre-testing*: A pilot study was conducted with 25 psoriasis patients. The mean comprehensibility score was 3.7 (on a 4-point scale), indicating good clarity. No modifications were necessary.
  The expert committee evaluated all items for cultural applicability beyond linguistic accuracy. No items required cultural modification, as the self-management domains assessed by the SMEQ-PSO were considered universally relevant across cultural contexts. This conclusion was further supported by the pilot study, in which all items were clearly understood without cultural misinterpretation (mean comprehensibility score: 3.7/4.0).
- *Step 6-Final version*: After completing all stages, the Turkish final scale version was created and submitted to the original developers for approval.

### Data analysis

Statistical analyses were performed using IBM SPSS Statistics (Version 26.0) and IBM SPSS Amos (Version 25.0). Descriptive statistics were expressed as frequencies, percentages, means, and standard deviations.

Content validity was assessed using the Davis technique [24]. Item-level content validity index (I-CVI ≥ 0.78) and scale-level content validity average (S-CVI/Ave ≥ 0.90) were calculated [25].

Construct validity was examined through EFA and CFA. Before EFA, Kaiser-Meyer-Olkin (KMO) and Bartlett’s tests were applied. Factors with eigenvalues greater than one were retained, and Varimax rotation was applied. CFA model fit was assessed using standard indices: χ²/df ≤ 3, RMSEA ≤ 0.08, CFI ≥ 0.90, GFI ≥ 0.85, TLI ≥ 0.90, SRMR ≤ 0.08, and factor loadings ≥ 0.30 [26, 27].

Reliability was evaluated using Cronbach’s α, McDonald’s Omega, and the split-half method. The acceptable value was ≥ 0.70 [28]. The significance level was set at *p* < 0.05.

## Results

### Characteristics of the psoriasis patients

A total of 147 patients participated in this study. The mean age was 45.16 ± 14.83 years, and 55.1% were male. The majority were married (70.7%), and 40.8% had university or higher education. The mean disease duration was 15.03 ± 13.25 years. The most frequently reported symptom was itching (72.1%), and the most common treatment method was the use of biological agents (55.8%) (Table 1).

**Table 1:** Descriptive characteristics of participants (*N* = 147)

Characteristic	Category	*n*	%
Gender	Female	66	44.9
	Male	81	55.1
Age	Under 35 years	41	27.9
	36–50 years	54	36.7
	Over 50 years	52	35.4
	***X ± SD***,*** M (min-max)***	45.16 ± 14.83, 44 (13–91)	
Marital Status	Married	104	70.7
	Single	43	29.3
Education Level	Primary education	36	24.5
	High school	51	34.7
	University	60	40.8
Employment Status	Yes	71	48.3
	No	76	51.7
Smoking	Yes	62	42.5
	No	85	57.5
Alcohol Use	Yes	24	16.4
	No	123	83.6
Disease Duration	10 years and under	70	47.6
	Over 10 years	77	52.4
	***X ± SD***,*** M (min-max)***	15.03 ± 13.25, 15 (1–60)	
PASI	***X ± SD***,*** M (min-max)***	1.97 ± 2.16, 1.2 (0–15)	
Experiencing Itching	Yes	106	72.1
	No	41	27.9
Experiencing Pain	Yes	44	29.9
	No	103	70.1
Treatment Method^İ^	Phototherapy	10	6.8
	Biological agent	82	55.8
	Systemic treatment	49	33.3
	Topical treatment	81	55.1

Note: PASI = Psoriasis Area and Severity Index; SD = standard deviation; M = mean. ⁱMultiple responses were permitted for treatment methods; percentages are not mutually exclusive and therefore exceed 100%

### Validity

#### Content validity

Expert opinions were evaluated using Davis’s four-point rating method [24]. I-CVI values for 28 items ranged from 0.86 to 1.00. The I-CVI was 1.00 for 22 items, indicating complete expert agreement. For six items (6th, 8th, 9th, 26th, and 28th), I-CVI was 0.86. All values exceeded the 0.78 threshold. The S-CVI/Ave was 0.97, above the recommended 0.90 threshold [25].

#### Construct validity

The KMO value of 0.92 and significant Bartlett’s test (χ² = 2854.79, *p* < 0.001) confirmed sampling adequacy for factor analysis. EFA revealed a five-factor structure explaining 70.5% of total variance: psychosocial adaptation (items 1–8), daily life management (items 9–16), skin management (items 17–21), disease knowledge management (items 22–25), and disease treatment management (items 26–28). Item-total correlations ranged from 0.47 to 0.74 (Table 2).

**Table 2:** Validity and reliability results for the self-management efficacy scale (*N* = 147)

Factor	Item Number	Factor Loadings	Total Correlation	Eigenvalue	Explained Variance %	Cronbach’s α	McDonald’s Omega
Psychosocial Adaptation	1	0.74	0.56	5.06	18.1	0.91	0.89
	2	0.79	0.71				
	3	0.73	0.66				
	4	0.71	0.68				
	5	0.66	0.58				
	6	0.73	0.67				
	7	0.64	0.66				
	8	0.73	0.58				
Daily Life Management	9	0.65	0.66	5.00	17.8	0.90	0.86
	10	0.60	0.60				
	11	0.68	0.60				
	12	0.80	0.63				
	13	0.74	0.53				
	14	0.81	0.67				
	15	0.72	0.55				
	16	0.57	0.65				
Skin Management	17	0.63	0.73	3.50	12.5	0.90	0.85
	18	0.70	0.74				
	19	0.77	0.66				
	20	0.70	0.67				
	21	0.71	0.60				
Disease Knowledge Management	22	0.76	0.67	3.12	11.1	0.92	0.82
	23	0.82	0.66				
	24	0.63	0.64				
	25	0.72	0.70				
Disease Treatment Management	26	0.80	0.47	3.05	10.9	0.91	0.89
	27	0.80	0.66				
	28	0.81	0.66				
Total	-	-	-	-	70.5	0.95	0.95

KMO = 0.92, D = 378, χ² = 2854.79, *p* < 0.001

Note: SD = standard deviation; M = mean

Although some items exhibited moderate factor loadings (e.g., Item 16 = 0.57, Item 9 = 0.65), all loadings exceeded the minimum 0.30 threshold. These items were retained for their theoretical relevance to the original five-factor structure and acceptable psychometric performance, consistent with the approach adopted in the original validation study (Sun et al., 2023). Item-total correlations for these items also remained within acceptable ranges (0.47–0.74), supporting their contribution to the overall scale.

CFA validated the five-factor structure with acceptable fit indices: χ²/df = 1.61; RMSEA = 0.07; CFI = 0.92; GFI = 0.88; TLI = 0.92; SRMR = 0.08; IFI = 0.93. All factor loadings were statistically significant (*p* < 0.05) (Table 3).

**Table 3:** Model fit indices for self-management efficacy scale (*N* = 147)

Scale	(χ²/df)	RMSEA	SRMR	IFI	CFI	GFI	TLI
Model	1.605	0.068	0.075	0.925	0.924	0.881	0.916

The GFI value of 0.88, while slightly below the conventional 0.90 threshold, falls within the acceptable range. GFI is known to be sensitive to sample size and model complexity, particularly in models with a larger number of estimated parameters relative to sample size (Sharma et al., 2005). The obtained GFI value is consistent with other Turkish psychometric validation studies employing similar sample sizes and structural equation models [26, 28], and the overall pattern of fit indices collectively supports adequate model fit.

### Reliability

#### Internal consistency

The scale demonstrated high internal consistency with Cronbach’s α = 0.95 and McDonald’s Omega = 0.95. Sub-dimension reliability coefficients were: psychosocial adaptation (α = 0.91), daily life management (α = 0.90), skin management (α = 0.90), disease knowledge management (α = 0.92), and disease treatment management (α = 0.91) (Table 2).

#### Split-half reliability

Split-half analysis yielded a Guttman coefficient of 0.97, with a correlation between halves of 0.95. Inter-subdimension correlations ranged from *r* = 0.35 to 0.65, and correlations with total score ranged from *r* = 0.68 to 0.84, supporting internal consistency (Table 4).

**Table 4:** Descriptive statistics and correlation matrix for SMEQ-PSO dimensions (*N* = 147)

	Statistics	1	2	3	4	5
**1**	**Psychosocial Adaptation**	**1**				
	*X ± SD*	18.89 ± 7.33				
	*M (min-max)*	18 (8–37)				
**2**	**Daily Life Management**	***r*** = 0.59*	**1**			
	*X ± SD*	19.65 ± 6.97				
	*M (min-max)*	19 (8–39)				
**3**	**Skin Management**	***r*** = 0.59*	***r*** = 0.63*	**1**		
	*X ± SD*	10.74 ± 4.45				
	*M (min-max)*	11 (5–23)				
**4**	**Disease Knowledge** **Management**	***r*** = 0.51*	***r*** = 0.54*	***r*** = 0.65*	**1**	
	*X ± SD*	8.45 ± 3.97				
	*M (min-max)*	8 (4–20)				
**5**	**Disease Treatment Management**	***r*** = 0.49*	***r*** = 0.35*	***r*** = 0.58*	***r*** = 0.65*	**1**
	*X ± SD*	5.45 ± 2.77				
	*M (min-max)*	5 (3–15)				
**6**	**SMEQ-PSO**	***r*** = 0.84*	***r*** = 0.83*	***r*** = 0.84*	***r*** = 0.78*	***r*** = 0.68*
	*X ± SD*	63.19 ± 20.68				
	*M (min-max)*	63 (28–131)				

Note: SMEQ-PSO = Self-Management Efficacy Questionnaire for Patients with

Psoriasis; SD = standard deviation; M = mean; r = Pearson correlation coefficient

*p < 0.05

## Discussion

This study conducted cultural adaptation and established the validity and reliability of the Turkish version of the Self-Management Efficacy Questionnaire for Patients with Psoriasis. The psychometric evaluation demonstrates that the adapted scale constitutes a valid, reliable, and culturally appropriate instrument for assessing self-management efficacy among Turkish patients with psoriasis.

The high content validity score (S-CVI/Ave = 0.97) indicates that the scale items were appropriately evaluated for their intended purpose and sensitive to the Turkish cultural context. This finding is consistent with the results from the original study and from scale adaptation studies across different chronic disease groups [26–28].

The KMO value of 0.92 strongly supported the sample’s suitability for factor analysis, slightly higher than the original study’s 0.91 [17]. The five-factor structure obtained from exploratory factor analysis largely overlaps with the original scale and supports cross-cultural consistency. The total variance ratio (70.5%) was higher than the 62.0% reported in the original study. The psychometric properties of the Turkish SMEQ-PSO are comparable to those of other adaptations of the Chronic Disease Self-Management Scale conducted in Turkey [26, 29].

Confirmatory factor analysis findings show that the five-factor structure is statistically confirmed in the Turkish sample. The χ²/df ratio of 1.61 demonstrates better model fit than the original study’s 2.42 [17]. The fit indices (RMSEA = 0.07, CFI = 0.92, GFI = 0.88) meet the commonly accepted thresholds in the psychometric literature and align with those reported in other Turkish validation studies [26, 28].

The overall Cronbach’s α of 0.95 exceeds the 0.93 reported in the original study [17]. This value is also comparable to other adaptations of the Turkish chronic disease self-management scale [26–29]. McDonald’s Omega coefficients were also calculated (0.95 for the total scale and 0.82–0.89 for sub-dimensions), indicating that both traditional and alternative reliability approaches support the scale’s internal consistency. Split-half reliability analysis yielded a Guttman coefficient of 0.97, consistent with the 0.95 reported in the original study [17].

The slight discrepancies between Cronbach’s alpha and McDonald’s Omega observed in certain subscales — most notably in Disease Knowledge Management (α = 0.92, ω = 0.82) — may reflect minor variations in factor loadings within these domains. Unlike Cronbach’s alpha, which assumes equal factor loadings across items, McDonald’s Omega accounts for such variation and may therefore yield a more conservative estimate. These differences are common in scales with multidimensional constructs. Importantly, both coefficients remained well above the 0.70 threshold across all subscales, supporting the internal consistency of the Turkish SMEQ-PSO.

The preservation of the five-dimensional structure in the Turkish sample supports the cross-cultural validity of SMEQ-PSO. As the SMEQ-PSO was recently developed (2023), the Turkish version represents one of the first cross-cultural adaptations of this instrument. The successful adaptation with improved psychometric properties suggests strong potential for international use. The limited availability of evaluation tools specific to psoriasis self-management skills in Turkey makes this adaptation valuable in clinical practice. The availability of culturally adapted instruments, such as the SMEQ-PSO, may facilitate future comparative research and support the evaluation of self-management outcomes across different healthcare settings.

While most existing psoriasis instruments — such as the Dermatology Life Quality Index (DLQI) for health-related quality of life and the Psoriasis Area and Severity Index (PASI) for clinical disease severity [30] — focus on disease impact or activity, tools that systematically assess self-management behaviors and efficacy are limited. The multidimensional SMEQ-PSO enables health professionals to identify where patients require support and to assess multiple aspects of self-management within a single instrument. Previous studies have demonstrated that nurse-led self-management interventions improve disease outcomes in patients with psoriasis [8, 12, 13]. In nursing practice, nurses who maintain sustained contact with patients with chronic conditions may use the SMEQ-PSO to support targeted care planning, individualized patient education, and longitudinal monitoring of self-management capacity. From a health services research perspective, the instrument provides a structured means of evaluating self-management efficacy as a service-related outcome in outpatient dermatological care. By enabling systematic assessment across psychosocial, behavioral, and treatment-related domains, the SMEQ-PSO may assist both clinicians and service managers in identifying patient groups requiring intensified education or follow-up, thereby facilitating more targeted and efficient service delivery. In addition, the scale may be used as an outcome measure for evaluating nursing-led self-management programs, outpatient education initiatives, and quality improvement interventions. At the service level, aggregated SMEQ-PSO data may contribute to monitoring outpatient care performance, informing resource allocation, and supporting evidence-based planning of chronic care services.

In routine clinical practice, the SMEQ-PSO may function as a practical screening tool during outpatient visits to identify specific self-management deficits. For example, low subscale scores in psychosocial adaptation may signal the need for referral to psychological support services or cognitive-behavioral counseling. In contrast, low scores in skin care self-efficacy may indicate that patients would benefit from structured skin care education and hands-on demonstration of topical treatment application. Similarly, deficits in disease knowledge may prompt nurses to implement targeted educational sessions covering disease pathophysiology, treatment rationale, and trigger-avoidance strategies. By identifying the specific domains in which individual patients report low self-management efficacy, nurses can shift from a generic educational approach to a tailored, patient-centered care plan. Furthermore, repeated administration of the SMEQ-PSO at subsequent clinic visits would allow longitudinal monitoring of self-management capacity, enabling nurses to evaluate intervention effectiveness and adjust care plans accordingly.

In addition to traditional nurse-led education, mobile health (mHealth) technologies and digital applications may serve as complementary tools to support sustained self-management in patients with psoriasis. Given the chronic and relapsing nature of the disease, such digital interventions may help reinforce self-management behaviors between clinical visits. Evidence from randomized clinical trials supports the efficacy of smart devices and mobile applications in improving health outcomes among patients with chronic conditions [31–33]. In this context, the SMEQ-PSO may be used as an outcome measure to evaluate the effectiveness of digital self-management interventions in future research.

Future studies should evaluate concurrent validity by examining correlations between SMEQ-PSO scores and established psoriasis-specific instruments, such as the Dermatology Life Quality Index (DLQI) and the Psoriasis Area and Severity Index (PASI), as well as generic self-efficacy measures, to provide additional evidence of construct validity.

### Limitations

This study has several limitations that should be acknowledged. The single-center design and the recruitment of patients from a university hospital dermatology clinic may limit the external validity of the findings, as these patients may not fully represent the broader population of individuals with psoriasis across diverse healthcare settings in Turkey. In addition, the sample predominantly comprised patients with mild disease activity (mean PASI = 1.97 ± 2.16), reflecting the characteristics of the outpatient population, which limits the generalizability of the findings to patients with moderate-to-severe psoriasis who may face distinct self-management challenges. Regarding sample size, although the number of participants (*n* = 147) met the minimum 5:1 participant-to-item ratio recommended for factor analysis, a more stringent 10:1 ratio (*n* = 280) would have provided greater stability in the factor structure. Furthermore, test-retest reliability was not assessed due to logistical constraints, as many patients attend dermatology clinics irregularly, making it difficult to schedule a second administration within the recommended 2–4-week interval. Similarly, concurrent validity was not evaluated; future studies should examine correlations between SMEQ-PSO scores and established instruments, such as the DLQI and PASI, to provide evidence of construct validity. Overall, future multi-center studies with larger, more heterogeneous samples — including patients across the full spectrum of disease severity — are recommended. Despite these limitations, the rigorous six-step adaptation methodology, comprehensive psychometric evaluation, and strong reliability indicators (α = 0.95, S-CVI/Ave = 0.97) support the overall validity and reliability of the Turkish SMEQ-PSO.

## Conclusions

The Turkish version of the SMEQ-PSO is a valid and reliable instrument for assessing self-management efficacy among patients with psoriasis. The scale retained its original five-factor structure with high content validity (S-CVI/Ave = 0.97), accounting for 70.5% of the total variance. Reliability analysis revealed high internal consistency (Cronbach’s α = 0.95, McDonald’s Omega = 0.95). The scale enables nurses to identify areas where patients need support and to develop individualized care plans. Beyond individual patient assessment, the scale may contribute to health service evaluation by enabling systematic monitoring of self-management outcomes in outpatient dermatological care settings.

## Data Availability

The datasets used and/or analyzed during the current study are available from the corresponding author on reasonable request.

## Abbreviations

CFA: Confirmatory Factor Analysis
CFI: Comparative Fit Index
EFA: Exploratory Factor Analysis
GFI: Goodness-of-Fit Index
I-CVI: Item-level Content Validity Index
IFI: Incremental Fit Index
KMO: Kaiser-Meyer-Olkin
PASI: Psoriasis Area and Severity Index
PSO: Psoriasis
RMSEA: Root Mean Square Error of Approximation
S-CVI/Ave: Scale-level Content Validity Index/Average
SMEQ-PSO: Self-Management Efficacy Questionnaire for Patients with Psoriasis
SRMR: Standardized Root Mean Square Residual
STROBE: Strengthening the Reporting of Observational Studies in Epidemiology
TLI: Tucker-Lewis Index
χ²/df: Chi-square/degrees of freedom ratio
